# Optimizing the Gut Microbiota for Individualized Performance Development in Elite Athletes

**DOI:** 10.3390/biology12121491

**Published:** 2023-12-05

**Authors:** Svenja Nolte, Karsten Krüger, Claudia Lenz, Karen Zentgraf

**Affiliations:** 1 Department of Exercise Physiology and Sports Therapy, Institute of Sports Science, University of Giessen, 35394 Giessen, Germany; karsten.krueger@sport.uni-giessen.de (K.K.); claudia.lenz@sport.uni-giessen.de (C.L.); 2 Department 5: Psychology & Sports Sciences, Institute for Sports Sciences, Goethe University Frankfurt, 60323 Frankfurt am Main, Germany; zentgraf@sport.uni-frankfurt.de

**Keywords:** human gut microbiota, performance, elite sport, exercise metabolism, individualization

## Abstract

**Simple Summary:**

The gut microbiota is fundamental for human health. This complex health construct is a pivotal part of performance development in athletes. The gut microbiota plays its role in the physiology of the athlete by influencing immune function, gut barrier integrity, macro- and micronutrient absorption, muscular efficiency, and the gut-brain-axis. This article displays possible ways in which the gut microbiota affects the performance of athletes and shows practical methods to improve athletes’ health and potential performance through a gut-centric approach.

**Abstract:**

The human gut microbiota can be compared to a fingerprint due to its uniqueness, hosting trillions of living organisms. Taking a sport-centric perspective, the gut microbiota might represent a physiological system that relates to health aspects as well as individualized performance in athletes. The athletes’ physiology has adapted to their exceptional lifestyle over the years, including the diversity and taxonomy of the microbiota. The gut microbiota is influenced by several physiological parameters and requires a highly individual and complex approach to unravel the linkage between performance and the microbial community. This approach has been taken in this review, highlighting the functions that the microbial community performs in sports, naming gut-centered targets, and aiming for both a healthy and sustainable athlete and performance development. With this article, we try to consider whether initiating a microbiota analysis is practicable and could add value in elite sport, and what possibilities it holds when influenced through a variety of interventions. The aim is to support enabling a well-rounded and sustainable athlete and establish a new methodology in elite sport.

## 1. The Human Gut Microbiota

Over the past several years, the human gut microbiota has become more and more popular in research and has been found to play a vital role in the physiological and psychological health of humans [1]. The analysis of the gut microbiota became more accessible through the non-invasive 16S ribosomal RNA (rRNA) analysis of small stool samples resulting in the collection of much more scientific knowledge [1]. The gut microbiota is defined as a collection of all symbiotic, commensal, or pathogenic microorganisms, including bacteria, archaea, fungi, and viruses, that live in the gut [2]. The entirety of microbes is called ‘microbiota’, whereas the term ‘microbiome’ includes the collection of genomes [3]. Research shows the importance of the microbiota to the physiology of the host, with key functions in the maintenance of the gastrointestinal barrier [3] supporting the development of the immune system and gut-associated lymphatic tissue [4] as well as metabolic functions, such as the availability of nutrients and energy homeostasis [5]. Two thirds of our gut bacteria are completely unique to each one of us and only one third exists in all of us [6]. Accordingly, scientists are trying to define a core microbiota that is identical and vital to all humans. The reason that this has not been achieved yet may be due to the theory that some bacteria can take over the central functions of others and are therefore interchangeable [7].

This article provides a comprehensive overview of the interaction between gut microbiota and sport, outlining potential influencing factors and deriving practical implications for athletes.

### Exercise as a Driver of Change in the Gut Microbiota

The gut microbiota is influenced by several factors such as genetics, mode of birth, medication, and lifestyle factors. One lifestyle factor that is supposedly a driver of change to the consistency of the intestinal microbiota is exercise [8]. Through cross-sectional studies, the first evidence of exercise affecting the shape of the gut microbiota emerged [8,9,10,11,12]. These differences were observed when untrained individuals were compared to individuals that exercise and who showed a higher microbial diversity. Additionally, intervention studies using physical activity in healthy sedentary participants and in populations with health disorders (e.g., inflammatory diseases, age-related pathologies) showed the beneficial effects of exercise on the gut microbiota [13,14,15]. 

## 2. The Gut Microbiota as Part of Individualized Performance Development in Athletes

Individualization is increasingly considered a potential performance reserve in elite sports, with measures aimed at enhancing performance development even for those already performing at a high level [16]. The structure of these factors is aligned with various models, such as Ullén et al.’s multifactorial gene–environment interaction model [17]. This model suggests that deliberate training impacts not only neural mechanisms but also physical properties. Baker et al.’s model [16] also fits well, as it considers not only training characteristics but also ability traits, the athlete’s physical attributes, dynamic interactions of influencing factors, and environmental and contextual conditions.

Recent studies indicate a high likelihood that the gut microbiota is among the significant physiological systems involved in individual performance development. This highly individual ecosystem has garnered increasing interest as a key player in human health and exhibits a bidirectional connection with physical activity [18]. These findings qualify the gut microbiota as an intriguing factor capable of influencing athletes’ performance on an individual basis.

Surprisingly, the analysis of the gut microbiota has been largely overlooked in the diagnosis and analysis of athletes. This oversight may be due to the lingering uncertainty about the role of the gut microbiota in the unique population of athletes and its potential added value to them. Additionally, its integration may be constrained by practicality issues for coaches and athletes as well as associated costs. Nevertheless, incorporating microbiota analysis may offer essential insights into health issues and specialized optimization strategies through nutrition and training.

### 2.1. The Link between the Microbiota and Athlete Physiology

The gut microbiota of athletes differs from that of untrained individuals, as it adapts to their extraordinary lifestyle with chronic training routines and probably differing dietary intake patterns [19]. More specifically, changes in the gut microbiota can occur due to the production of short-chain fatty acids (SCFAs) [20,21], potential weight loss [22], alteration of the bile acid profile [23], modulation of the immune system, such as immunoglobin A [24], the number of T and B cells [22] and changes in toll-like receptor (TLR) signaling pathways [25,26]. The athletes’ gut shows an increased abundance in *Bacteriodetes*, *Prevotella*, *Methanobrevibacter*, *Akkermansia* [27], and *Akkermansia muciniphila*, which could positively influence the mucosa layer [8,28]. Additionally, a connection of the maximal oxygen intake (VO_2_max) and the intestinal microbiota has been proposed in some studies, suggesting that VO_2_max positively correlates with the gut microbiota, more specifically with the metabolic functions of the bacteria [29,30]. The work of Estaki et al. showed that cardiorespiratory fitness correlates with an increase in the diversity of the microbial community and that these results are centered around functions more than taxa [29]. Likewise, the F/B ratio has been suggested to be, when increased, associated with a higher VO_2_max [30].

It has also been suggested that the composition of the gut microbiota can vary between different sport modalities. Only a few studies have investigated these differences. The work of Mohr et al. [31] and Aya et al. [32] summarized that in most cases, alpha and beta diversity show no differences between sport modalities. One study in Irish elite athletes found variances in the microbial profile between different sport modalities. The study collected data from 37 individuals across 16 disciplines [33]. A recent study supports this data, showing that different types of exercise have different effects in female athletes but not in male athletes. Li et al. more specifically investigated differentiating inflammation patterns in varying exercise modalities, concluding that different types of exercise cause different inflammation patterns [34]. These discoveries suggest that some bacteria may favor a certain sport. The controversial evidence once again highlights the difficulty in defining the gut microbiota, most likely due to its interaction with all other physiological systems of the individual’s body. Nevertheless, the above discussed results of recent literature highlight the potential linkage between the intestinal microbiota and performance and indicate a symbiotic relationship in the adaptations of these physiological systems. What shape this connection unfolds to look like is not clear to date, which mirrors the aim of this review to evaluate all aspects that could be relevant to an athlete performing at an elite level, consistently based around the aim of building a healthy and sustainable athlete. 

#### 2.1.1. Bioavailability of Substrates, Micro- and Macronutrients

The microbiota breaks down substrates and produces vitamins and several signaling molecules. Some authors go as far as saying that the microbiota acts like an endocrine organ and has the power to improve metabolic pathways and the immune capacity [35,36,37]. For athletes, it is essential to have an adequate dietary strategy to support their high training load, involving a much higher requirement for both of macro- and some micronutrients [38]. Finding the right balance between efficient and quick fueling and a long-term approach for well-rounded athletes, including a performance-supporting gut microbiota, is complex. The next chapters will attempt to act as a guide through the abundance of ways in which the microbiota influences the bioavailability of substrates and micro- and macronutrients in an athlete’s gut. 

##### Carbohydrates

Carbohydrates can be classified into those that are digestible and those that are non-digestible. Digestible carbohydrates are metabolized in the small intestine through enzyme breakdown. Non-digestible carbohydrates are fermented in the large intestine by microorganisms [1]. For athletes, highly digestible and energy rich carbohydrates are the key to maintaining glucose homeostasis, help performance, and reduce fatigue [39,40]. On the flip side, evidence shows that a diet dominantly low in fiber and high in digestible carbohydrates is detrimental to gut health, causing the loss of microbial diversity, damaging gut integrity, and decreasing SCFA production [41,42,43]. SCFAs are produced during the breakdown of indigestible structures and can, through oxidation in the skeletal muscle, help maintain performance by increasing the bioavailability of fatty acids, glucose, and glycogen [44]. They are primarily important in endurance events, where one of the limiting factors is glycogen storage [45]. Acetate, propionate, and butyrate are the primarily produced SCFAs. Bacteria that are known to produce SCFAs are *Bacteroides*, *Bifidobacterium*, *Propriobacterium*, *Eubacterium*, *Lactobacillus*, *Clostridioides*, *Roseburia* and *Preovotella* [46].

Accordingly, a proper fueling strategy that on the one hand benefits performance momentarily and on the hand supports athletes overall gut health needs to be accomplished.

##### Protein

Protein is another key macronutrient in an athlete’s diet. Through a much higher energy production for gut integrity and protein synthesis, protein intake is increased in a trained, compared to an untrained individual [47]. Other than strength training, especially long endurance sessions must be fueled with an additional source of protein, as myofilament proteolysis may increase [48,49,50]. Around ten percent of protein cannot be digested and is fermented by microbes [51,52,53], producing metabolites like SCFAs and branched chain amino acids (BCAAs), that can have favorable effects on gut integrity and performance [51,54]. Furthermore, research suggests that the microbiota influences muscle anabolism and functionality, indicating the existence of the gut-muscle-axis [55,56,57,58]. The gut microbes influence proteolysis, by producing peptides and proteinase that work alongside human ones [59,60]. Protein consumption also positively correlates with microbial diversity [7]. The quality and quantity of protein intake must be considered, especially when the consumption is higher due to an intense training routine. Firstly, there is the choice between protein from whole foods or a supplement. Whole foods seem to have an equal or an even better ergogenic effect, as it is suggested that other components like fat are instrumental in protein synthesis [61,62]. Further decisions must be made regarding the source of the protein, between plants and animals. Animal protein seems to increase the abundance of *Bacteroides*, *Alistipes*, *Bilphila*, *Ruminococcus* and decrease the abundance of *Bifidobacterium* and SCFA production compared to a plant-based protein, which additionally increases the abundance of *Bifidobacterium* and *Lactobacillus* and decreases the abundance of *Bacteroides* and *Clostridioides*. Some research even suggests that animal protein can have a negative effect on the gut microbiota, caused by its harmful by-products [63]. A combination of a protein supplement and a probiotic could be worth considering. The study of [64] showed that *Bacillus coagulans* taken with protein, decreased inflammation, improved nutrition absorption and increased the production of proteases that improve amino acid absorption. This may lead to a reduction in muscle damage and improved recovery and therefore may favor performance enhancement [65].

Choosing the source of an athlete’s protein is one thing, finding the right amount is another. The balance of supporting the training load and the ongoing adaptation processes while also not overloading the system is needed. An immoderate digestion of protein can cause distress to the host and have detrimental effects on gut permeability and lead to higher levels of inflammation, due to an increase in proteolytic metabolites [51,52,54,66,67,68]. Overall, adequate protein intake can be beneficial for microbial diversity and is fundamental for athletes’ health and performance.

##### Fat

Fat is essential for a well-functioning human body, as it is part of the cell-membrane, a source of energy, and allows the absorption of fat-soluble vitamins [69]. During exercise, free fatty acids, adipose tissue, and triglycerides provide a generous source of energy, which becomes even more important in endurance events [69]. Regarding the gut microbiota, research shows that fat has the power to change the intestinal microbiota and support athletic performance, therefore it is important to choose the type and quantity wisely [53,69]. Apart from using fat as fuel in endurance events, there is rising interest in a ketogenic diet which focuses on high fat and low carbohydrate foods [70]. However, the promise of supporting performance could not be proven in research. In fact, some studies have shown that this way of eating could even lower performance in high intensities [69,70,71,72]. Focusing on the microbiota, a high-fat diet in rats showed a decrease in *Lactobacillus* and an increase in *Clostridiales*, *Bacteroides and Enterobacteriales* [7]. There is also an indirect effect from a high-fat diet and higher bile acid production, which leads to an acidic environment that is antimicrobial [73]. Whereas secondary bile acid, produced by the microbiota seems to improve energy and oxygen absorption, through a higher oxidative phosphorylation and ß-oxidation of fatty acids, leaving athletes more resistant to fatigue [74]. The microbiota seems to have the power to modulate bile acid production and may modulate performance in athletes [21,56]. As mentioned above, the type of fat must be considered as well. Saturated fats can increase *Bacteroides, Bilophila,* and *Faecalbacterium prausnitzii* [7] and show a higher increase in lipopolysaccharides (LPS) than unsaturated fats in a mice model [75]. Unsaturated fats can increase *Streptococcus*, *Lactobacillus*, *Bifidobacterium*, and *A. muciniphila* [7]. Furthermore, Omega-3 fatty acids increase SCFA production, gut integrity, and may influence the gut-brain-axis [76]. 

Altogether fat is an energy provider of the athlete’s diet and can, when used in the right form, positively modulate the intestinal microbial community by an increase in favorable bacteria. 

##### Micronutrients

Micronutrients are essential for human health, supporting immune function, bone health, and metabolism [77]. The need for micronutrients is due to heavy training loads, excessive sweating, and higher oxidative stress in athletes than in untrained individuals [77,78]. A deficiency in nutrients can impact the gut microbiota and in some cases vice versa [79].

##### Vitamins

Vitamins are micronutrients that are essential for the physiological functioning of the human body and function as cofactors for enzymes [4]. Being deficient in a vitamin can cause chronic health issues, while on the other hand, consuming multi vitamin supplements can alter the gut microbiota, because only small amounts can be absorbed in the upper gut [80]. For athletes, the adequate supply of antioxidative vitamins, such as vitamin C and E, plays a crucial role in the reduction of oxidative stress caused by exercise. Research on piglets showed the reduction of free radicals and increasing *Bifidobacterium* and *Lactobacillus* and reducing *Escheria coli* [81]. Looking at this from an athlete’s perspective, antioxidative, but also anti-inflammatory vitamins, such as vitamin D, might help immune function and also promote the microbial microenvironment [38]. The study of Singh et al. [82] showed a dose-dependent increase in vitamin D-deficient subjects of the relative abundance of *Bacteroides* and *Akkermansia*, a decrease in the *Firmicutes*/*Bacteroidetes* ratio and a decrease in the relative abundance of *Faecalibacterium* and *Ruminococcaceae* in the responder group. Furthermore, *Bacteroides acidifaciens*, *Ruminococcus bromii*, *Bacteroides eggerthii*, and *Barnesiella intestinihominis* showed a significant enrichment in both pre- and post-supplementation in the responder group. These findings suggest that an enrichment of these bacteria could be associated with a response to vitamin D supplementation. On top of that, the bidirectional interaction between the microbiota in inflammation and the vitamin D status, leads to a hypothesis, that the make-up of the microbiota may affect vitamin D intake [82].

##### Minerals and Trace Elements

Minerals and trace elements are also vital micronutrients for human metabolism and interaction with the microbial community [83,84]. When focusing on those that are relevant for performance and stand out due to deficiencies, calcium is a key element in an athlete’s diet, mainly because it is important for bone health and muscle contraction [85]. Particularly female athletes with an exhaustive training load are at risk of calcium deficiency and developing symptoms of relative energy deficiency in sport (REDs) [86]. Even though calcium can easily be consumed in adequate amounts in an omnivore diet, it is not uncommon for it to be deficient. Furthermore, even an energy deficiency of five days is enough to increase bone formation in both men and women [87,88]. A study in pubertal children showed that a moderate daily consumption of maize fiber for a period of three weeks increased calcium absorption, which goes hand in hand with a shift in the gut biome increasing *Bacteroidetes* and decreasing *Firmicutes* abundance [89]. Further research shows an increase in SCFA lowers the pH in the colon which helps mineral solubility, resulting in a better calcium absorption. The gut biome seems to influence both bone modulation and formation [90,91]. Zinc deficiency is rather common in athletes and leads to disordered eating with a drop in endurance capacity, weight loss, and fatigue [92]. High-risk groups have been identified as endurance athletes that have a high carbohydrate but lower protein and fat intake [92], female athletes [93], athletes with a low calorie intake [94], and vegan athletes [95]. Research is limited in terms of human studies that analyzed the connection between zinc and the gut microbiota. Animal studies show a potential interplay between the microbiota and zinc status [96,97], showing that the supplementation of a prebiotic led to the higher presence of *Bifidobacterium* and *Lactobacillus* [96]. Another model demonstrates the positive effect of zinc-enriched probiotics on immune function in animals [97], while on the other hand excessive zinc intake can have negative effects on the gut [98]. Iron is important for oxygen transport and energy metabolism [99]. 

Iron deficiency is common in athletes [99,100]. Oral iron supplementation is often associated with nausea, gastric irritation, and other symptoms [101], while further supplementing with iron reduces the abundance of lactic acid bacteria and increases the enteropathogenic *E. coli* [102]. Absorption can be achieved naturally in a diet high in unprocessed foods [103] and phytate reduction may help absorption [104]. The review and meta-analysis of Vonderheid et al. (2019) [105] reports that probiotics seem to positively influence iron absorption, especially *Lactiplantibacillus plantarum* 299v significantly increased the absorption of non-heme iron [105]. Genetics are a factor that is shown to highly influence iron status and may also be the cause of poor absorption [106]. The last one to touch on is magnesium, where the requirement can be up to twenty percent higher in athletes [107]. Again, there is a distinct lack of evidence from human studies, although animal studies include good evidence showing the interplay between magnesium absorption and the composition of the gut microbiota [108,109,110]. 

##### Metabolic Intermediates

One study revealed a direct connection between lactate metabolism and gut microbes. In this study Scheiman et al. showed that lactate is able to pass the gut lumen and can be metabolized into propionate by the *Veillonella* bacteria [27]. Furthermore, due to training, the abundance of *Veillonella* can increase. This was presented by an investigation on marathon runners participating in the Boston marathon in 2015, showing an increase in the abundance of *Veillonella atypica* after the race [27]. Mice that were implanted with *Veillonella* showed better performance on the treadmill than controls. These findings demonstrate the ability of the microbiota to change metabolism and modulate SCFA production, which can then be used in hepatocytes and muscle cells to maintain glucose homeostasis [57]. A lactate rich environment facilitates the proliferation of *Veillonella atypica* and seems to be linked to improved running times [27]. 

A recent study by Fontana et al. (2023) analyzed the metagenomic and metabolomic profiles of athletes, moderate athletes, and sedentary individuals and found major differences in the enzymatic functional clusters (EFC) of bacteria in athletes compared to sedentary individuals [111]. The EFC related to athletes was positively linked to 73 high biological impact synthases (HBIS) and 742 enzymes, whereas the EFC related to sedentary individuals was positively linked to 14 HBIS and 105 enzymes. This highlights how the extraordinary lifestyle of athletes pressures the gut microbiota to reshape and adapt to bacterial species with a higher enzymatic capacity, impacting the host’s health and muscular performance. Additionally, SCFA producing microbes are suggested to have a much wider range in the production of functional impact molecules [111].

#### 2.1.2. Gut Barrier Integrity

Gastrointestinal problems are a common problem in sport, especially in endurance sports [39,112]. Therefore, gut barrier function plays a pivotal role in the performance of elite athletes. The gut barrier is especially important in keeping pathogenic microorganisms separate from other sterile environments and organs [1]. SCFAs are known to support gut barrier integrity [7], just as it has been shown that bacterial bile acids are also important for barrier function [113]. With all the positive effects of training, there are some effects that can alter the gut microbiota and gut permeability [55,114]. Combined long-lasting and exhaustive endurance exercise increases gut permeability and decreases gut mucous thickness, letting potentially harmful microorganisms float into the blood stream, causing an inflammatory reaction [7]. In the blood, LPSs from the microbiota activate TLRs and activate the Nf-kb pathway and pro-inflammatory cytokines. These cytokines support the process of increasing gut permeability further, as they loosen intestinal tight-junctions [115]. This process is further enhanced through a hypoxic state, followed by a reperfusion, which “washes” bacteria in the sterile regions of the body [116]. On top of that, a hypoxia-reperfusion model in rats from Wang et al. showed that this process leaves a state of dysbiosis, with an overgrowth in *E. Coli* [117]. Another study in soldiers showed a shift from dominate phyla, like *Bacteroides*, to less dominate phyla [116]. These changes could further increase gut permeability and bacteria translocation. The microbiota took 72 h to recover completely, which shows the detrimental effect of excessive endurance events [117]. This leaves room to support the recovery process of the gut microbiota and may enhance performance.

Another component of training that alters the gut microbiota and changes gut permeability is the production of reactive oxidative species (ROS). These are produced during exercise and by some commensal bacteria of the microbial family [118,119]. The ROS produced by the microbiota seem to have a positive effect on other microbes on the gut and have modulating effects on the host organism, including the handling of ROS produced by exercise. The oxidative stress that occurs due to exercise can decrease or even hinder the modulating effects of the microbiota [119,120]. Therefore, training should always be aligned with training status—if not considered wisely, this can have detrimental effects on systemic inflammation, oxidative stress, muscle damage, and can decrease immune function [38].

#### 2.1.3. Muscular Efficiency

Another area of interest is the role that that the microbial community plays in muscular efficiency, enhancing the existence of the gut-muscle-axis [55,56,58]. That fact that there must be a direct connection between the gut biome and our skeletal muscle has been shown in a study where the microbial make-up helped to correctly predict people suffering from sarcopenia and physical fragility [121]. So, the connection is there, but what does that mean for an athlete that is trying to reach peak performance?

Evaluating athletes for indications of dysbiosis is crucial in this area of interest. Intestinal dysbiosis is associated with enormous metabolic modulations, such as inflammation, pro-anabolic mediators and protein synthesis, all leading to alterations in skeletal muscle physiology [55]. Synthesis and absorption of vitamins is a key component of a well-functioning microbiota, as discussed in Section 2.1. Some of those vitamins have pro-anabolic effects on the skeletal muscle, including amino acid synthesis and oxidative stress modulation during training [122]. The production of bile acids also plays an important role in the gut-muscle-axis, which increases the farneosid X receptor (FXR) inhibition. The receptor is involved in energy metabolic pathways, glucose, and lipoprotein turnover. By modulating this receptor, the microbiota plays a part in the metabolic balance and myocyte anabolism [121,123]. SCFA production influences muscle protein accumulation by regulating anabolic/catabolic equanimity [124]. The application of probiotics and butyrate has been proven to help muscle wasting in pre-clinical studies and potentially shows another path to enhance training adaptation and performance in athletes [125,126]. The link between the gut and skeletal muscle is bidirectional, showing that training affects the gut biome and ultimately impacts factors such as protein synthesis [75,127].

Further findings propose SCFA targets skeletal muscle that contains G-protein-coupled receptors (GPR41 & GPR43). These receptors are SCFA-specific receptors supposed to be involved in muscle metabolism. Findings such as these are of particular interest when thinking about the essential role of energy metabolism during exercise [128]. 

#### 2.1.4. Immune Function

The physical abilities of the body are put through some highly challenging tests during training and even more in competition and races; while trying to keep homeostasis and organs functioning, performance should not suffer. Especially during endurance events, the demands on the immune response of the body are high, often resulting in a higher occurrence of upper respiratory tract infections (URTIs) [126] and gastrointestinal (GI) problems [129]. The link between the gut and the host’s immune response has clearly been shown by the fact that a disruption of this connection can cause autoimmunity and several other inflammatory processes [7]. The cellular immune system is closely dependent on the gut microbiota because the gut tissue harbors gut-associated lymphoid tissue (GALT). It is estimated that in humans, about 70% of immune cells are functionally associated with GALT and thus contact with the gut microbiota is essential for the functioning of the host immune system [130,131]. It seems that this could be an important switch to flick in taking better control of an athlete’s performance. Considering the immune response of an athlete, there are two ways in which this can be decreased but also improved. One is the gut barrier, which we touched on in Section 2.1.2, whereby this “gate” indirectly modulates the immune system by keeping pathogens from colonizing in the body [7] and secondly, directly via metabolites like tryptophan, retinoic acid, and SCFA [132]. By itself, research regarding probiotics in sport can give limited information about recommendations and advantages [133]. Still there are studies showing that probiotics can modulate the body’s immune response in many ways, by inducing IL-10 production [133], T and B cell activation [134], and IFN-γ and IgA [135], and further inhibiting NK cell activity [134] and therefore may lead to a reduction in the severity and incidence in URTI. These effects seem to be strain specific to lactic acid producing bacteria: *Lactobacillus* and *Bifidobacterium* [1]. Studies in cyclists, elite rugby players, and long distance runners showed the positive impact of supplementation on the host’s health [136,137,138,139].

##### Gut Microbes for Damaged Muscles—A Mouse Model

A recent study revealed a population of ROR γ + Treg cells that accumulate after acute muscle damage. These cells have been shown to be regulated by the gut microbiota. Mice that were missing these colonic Treg cells had slower muscle recovery, increased inflammation, and developed scars and fibrosis. Mice that were given antibiotics to kill beneficial gut bacteria showed slower muscle recovery. Furthermore, ROR γ + Treg cells have been observed to suppress the inflammatory signal IL-17 and further support a balanced proliferation and differentiation of muscle stem cells. Additionally, the authors named the ROR γ + Treg cells as general keepers of homeostasis in extra-gut matters. The therapeutic use of antibiotics following a muscle injury should be reconsidered as it could interfere with the healing process. Studies like this open an exciting outlook into a potential treatment method using microbes for injured muscle. However, since the study was performed in a mouse model, it is emphasized that human studies are needed to prove this potential [140].

#### 2.1.5. The Gut-Brain Axis

The bi-directional communication between the brain and the gut is called the gut-brain axis [141]. The microbiota has been suggested to play a fundamental role by producing neurotransmitters and hormones that can affect athletes in terms of their perception of fatigue, motivation, and mood [114]. Research on humans is lacking in this field but mice models show that the microbial community directly and indirectly influences serotonin levels [141]. The direct connection is highlighted by the fact that germ-free mice have decreased serotonin levels [142] and increased tryptophan levels [143]. The connection between the enteric nervous system and the gut mainly goes through the vagus nerve [144], hormones [145], and microbial metabolites [146,147]. Furthermore, stress is an immense factor in the career of an athlete, including physiological and psychosocial stressors [148]. While stress is vital for adaptation in homeostasis and performance [148], it should not be overreaching coping mechanisms. Training causes a stress response via the hypothalamus-pitularity-adrenal axis (HPA) and sympatho-adrenomedullary axis (SMA) causing a release of several catecholamines and glucocorticoids [149]. If the training-induced stress is straining for the host organism, then damage to gut barrier integrity can occur, leading to the problems mentioned above [119,120]. Studies in mice show how the microbiota can function as a ‘buffer’ to these stress reactions. Germ-free mice responded with an exaggerated release of the adrenocorticotropin hormone (ACTH) and corticosterone in comparison to the pathogen-free control group [150]. The effects were shown as a two-way street in another mouse model. Allen et al. compared voluntary wheel running and forced wheel running over six weeks, presenting that markers for bowel disease and immune function were lower in the group of voluntary wheel running [20].

Even though these findings focus on animal models, they show a strong connection between the gut and the brain. As the microbiota is highly influenced by diet [151], this may again open up another powerful way to boost performance.

#### 2.1.6. Specific Aspects of Female Athlete Health

It has been reported that the gut microbiota, just like other physiological systems of the human body, is marked by sex differences [152]. For females in particular, it is suggested that hormonal fluctuations stress the make-up and function of the intestinal community [153]. It has already been suggested in previous years that there is a bi-directional connection between the gut microbiota and estrogen [154]. Gut microbes seem to be capable of the modification of estrogen, the synthesis of estrogen, and the expression of hydroxyl-steroid dehydrogenase [155]. Furthermore, the menstrual cycle brings on a variety of different symptoms, many of them being centered around the gastrointestinal tract, including symptoms such as nausea, diarrhea, and bloating [156]. These symptoms and general gastrointestinal differences, such as transit time, could impact the food and nutrition strategy of the athlete and furthermore alter the training routine. A large study including 1812 eumenorrheic women that were recruited by the Strava app reported that menstrual symptoms were associated with a greater likelihood of missing a sporting event and missing or changing training [157]. 

Another aspect of athlete health is adequate energy availability to secure overall health and beyond that, allow performance [158]. Women as well as men are at risk of developing REDs. As this chapter focusses on the female athlete, this will be the emphasis further in this chapter. REDs can lead to variety of symptoms, from GI problems to lowered bone mineral density [159]. This indicates an involvement of the gut microbiota and the literature provides hints that could support this connection. Firstly, studies focusing on the eating disorder anorexia nervosa (AN) report a decrease in bacterial families, species, and genera within the *Firmicutes* phylum [160,161]. The genera *Clostridioides*, *Roseburia*, and *Ruminococcus* are reported to be decreasing as well. These genera are known for their carbohydrate fermentation properties and are major producers of SCFAs [162]. SCFAs on the other hand are key modulators in the regulation of gut homeostasis [163]. Additionally, studies established that SCFAs are regulators of bone metabolism and, more specifically, osteocyte metabolism while some SCFAs inhibit osteoclast differentiation and can reduce bone absorption [164]. Different intestinal microbiota have been shown to affect nutrient intake, such as elevated levels of *Lactobacillus* and *Bifidobacteriuml* which can promote the absorption of some minerals (calcium, magnesium, and phosphorus) that increase bone mineral density [165]. There is a lack of literature regarding this topic, but there are some indications that the gut microbiota has an important function in energy availability and bone metabolism in female athletes.

## 3. Targeting Performance Enhancement in Athletes

**Figure 1:**
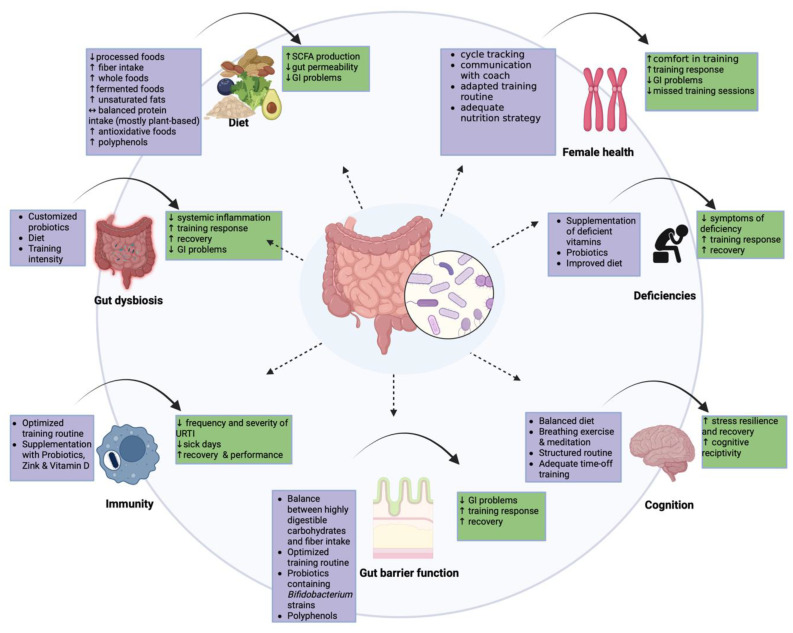
Intestinal health as a key pillar for performance enhancement in elite athletes. Six targets have been described, including gut dysbiosis, diet, deficiencies, immunity, gut barrier function, and cognition. All targets are highly connected and are centered around the gut microbiota. The purple boxes display the intervention that should be considered if a problem is identified in this particular area. The green boxes show potential positive outcomes for the athlete following the suggested intervention. All six areas can positively affect each other via the microbiota when there are no concerns in these regions or the interventions are shown to be effective. Picking diet as an example, it influences all of the other targets and most of those connections are bi-directional. Through a balanced diet, metabolites such as SCFAs are produced by the microbial community and positively influence immunity and gut barrier function and can further improve the symptoms of dysbiosis. Diet has also been shown to affect stress response and have a corresponding effect via the gut-brain axis. A wholesome diet also covers micronutrient intake to a high extent. When an area is recognized as problematic, it can greatly affect other areas as well, as they are connected through the gut microbiota. For example, gut dysbiosis can lead to increased gut permeability decreasing stress resilience and recovery, adequate nutrient absorption, and altered immunity through a systemic low-grade inflammation. Created with BioRender.com.

We believe, backed up by a growing body of evidence, that there is a potential for the gut microbiota to be a variable that could be beneficial for the performance of athletes and further lead to a healthier and therefore more sustainable athlete, since another part of human health is included with the gut microbiota. Consequently, we recommend starting to include gut microbiota analysis in performance diagnostics (Section 4). Though the microbiota is resilient and is fairly stable, it undergoes changes due to variations like deviating diet or training intensity [166]. A recent work by Akazawa et al. showed a change in the gut microbiota at the genus level, when comparing the preparation and transition period of a season [167]. Another study by Karl et al. looked into military personnel performing high-intensity interval training resulting in a significantly changed gut microbiota composition after a period of four days [168]. Having check-ups shows how the microbial community responds to routine changes and ensures the ongoing health of a sustainable athlete, even beyond their career (Figure 1). The following targets have been put together with the aim of enhancing performance in athletes in a way that is sustainable. All six targets are in some way linked or influenced by each other. The approach is multifactorial, complex and invites us to take a broader, more holistic look at athletes.

### 3.1. Targeting Dysbiosis

The first step of the analysis of the stool is to check if there are indicators for dysbiosis. Indicators can easily be found through questionnaires asking for symptoms such as altered gut transit time, diarrhea, and discomfort [169]. Further questions regarding the diet and training routine help provide a clearer picture. A combination of both stool analysis and a catalog of questionnaires is essential. As mentioned previously, high amounts of monosaccharides and low amounts of fiber can enhance gut dysbiosis in athletes [114,170,171] which, topped off with strenuous physical and psychosocial effectors and regular ischemia reperfusion through high volume endurance training, can lead to an imbalanced microbial community [112,172,173]. With the 16sRNA of the stool, alterations in diversity and metabolic capacity, increased pathogenic bacteria, and the loss of the main taxa can be detected. Corresponding results would lead to interventions, including customized probiotics to increase diversity and abundance of main taxa, potentially leading to a decrease in systemic inflammation and GI problems, as well as the targeting of the diet, leading to the next target point and further evaluation of the health status of an athlete, including deficiencies, immunity, barrier function, and cognition [174].

### 3.2. Targeting the Diet

Diet is a key modulator for the gut microbiota and can act as a diagnostic tool in the assessment of the dietetic status using questionnaires and software to review micro- and macronutrient intake. Accordingly, it serves as a practical instrument in individualized performance development.

Diet and training are often hard to combine, as it is difficult to obtain the right food and vitamins in between training sessions, hence why infrastructure and education play a central part in this. Awareness of the importance of diet and correct fueling should start with young athletes. Educating trainers, parents, and other people involved, such as the employees of boarding houses, is also vital. Diet is highly individual [175] and fueling strategies must be individualized, emphasizing the importance for every athlete to find their own way that works. 

Diet is a fundamental component for the composition of the microbiota [151] and fueling techniques should include gut health. Carbohydrates can and should come, in some situations, in highly digestible versions. In an approach that considers the microbial community, fueling strategies must be chosen more wisely. This could just be by ensuring that beyond training, there is adequate fiber in fermented food and a polyphenol-rich diet that potentially helps buffer those harmful effects to some extent [44,176,177,178]. Furthermore, a fiber rich diet may even support performance through higher SCFA production [38]. Fiber should adequately be increased in accordance with the higher caloric intake of athletes. The German nutrition society (DGE—Deutsche Gesellschaft für Ernährung) recommends a fiber intake of 14.6 g/1000 kcal for adults. For an athlete that consumes between 3000–4000 kcal per day, a fiber amount of at least 43.8–58.4 g of fiber would be appropriate, not including outbalancing highly digestible carbohydrates. Fueling with lactose is a technique of combining fueling and supporting gut health. Research suggests it is a good fueling source for before, during, and after practice, increasing performance and recovery, and additionally it may even promote the higher abundance of *Lactobacillus* and *Bifidobacterium* [179]. This, just like all other recommendations, needs to be tested as there can be great interindividual differences.

Protein is the thing that comes into an athlete’s mind first when speaking about deficiencies and supplementation. This is to a high extent caused by the media attention surrounding protein supplements and protein-rich products [180]. The impact that protein has on the microbiota has already been discussed above. The amount of protein is an important variable; excessive protein intake can have detrimental consequences for the microbiota and overall health of the athlete [51,52,54,66,67,68]. Evidence suggests that high protein intake must come with an adequate amount of fiber, which can help reduce the harmful effects on the biome [181]. Having whole food sources can be suggested, as compounds like fat help absorption and are beneficial for the gut biome [61]. 

Fermented foods have gained popularity through their promising anti-inflammatory properties [177,178] and the positive modulation of the microbial community [182]. Human dietary studies show improved glucose metabolism and a decrease in muscle soreness when resistance training was followed by fermented milk [177]. A study by Sonnenburg et al. [151] highlights these effects indicating a decrease in all inflammation markers in the intervention group that consumed fermented foods, including IL-6, IL-10, and IL-12b and other inflammatory factors [183]. Adding fermented foods to a whole diet could benefit an athlete and the inflammatory processes caused by a high training load. 

Another food constituent worth considering is polyphenols. Exhaustive training leads to a high load of ROS and causes fatigue, muscle weakness, and injuries in athletes [184]. Polyphenols have been recognized for their antioxidative properties. Polyphenols are plant-derived compounds that can be found in vegetables, seeds, fruits, wine, tea, coffee, and cacao [7], often taken in a supplement form [185]. Polyphenols not only directly influence the host by eliminating ROS, but also by modulating the gut microbiota [186], increasing commensal bacteria, such as *Lactobacillus* and *Bifidobacterium*, and working against pathogenic species [7]. The microbiota is responsible for the absorption and production of more bioavailable metabolites of polyphenols [185,186]. Although the gut biome is able to transform polyphenols into a more bioavailable form, many natural polyphenols, like those bound with sugars, have a low absorption rate in the human gut [187]. Therefore, those effects may be limited due to absorption, but also once again highlighting the role of gut biome modulating compounds in a way that they become more beneficial for an athlete’s metabolism. A wholesome diet, rich with a variety of fruit and vegetables (preferably seasonal) should be able to provide these benefits to the gut biome. Further supporting it with other central compounds such as fiber and fermented foods could add some valuable effects.

### 3.3. Targeting Deficiencies

A sufficient vitamin and mineral status is vital for performance [188]. Therefore, regular check-ups in commonly deficient and performance-related vitamins and minerals should be made. When the microbial profile and diet cannot be identified as the cause of these deficiencies, the specific shape of the bacterial community can be analyzed. Research has named bacteria that are linked to mineral bioaccessibility and bioavailability.

It might therefore be useful to see if a deficient mineral is also associated with a less abundant bacteria strain that has been identified as a variable in controlling the absorption of that vitamin or mineral. This becomes even more important when the athlete is not responding to high-dose supplementation. This is exactly what was shown in the work of Singh et al., (2020) [82] who found that the non-responder group to vitamin D had lower levels of *Bacteroides acidifaciens*, suggesting that the gut microbial profile is linked to vitamin absorption by either supporting or hindering it. Supplementation with the right probiotic could eventually help with this problem when diet is not the limiting factor.

### 3.4. Targeting Immunity

Sick days are something that can get in the way of high performance, especially when training for a season highlight, or even worse, a career highlight. Therefore, limiting time spent sick is another target on the road to reach peak performance. The gut microbiota has been identified to play a key role in our immunity [127,128,189]. Immune function is highly dependent on gut barrier function (further discussed in 3.5.), as when the gut permeability rises, it opens up like a “gate” and pathogens can exit into the blood stream, causing inflammation [7]. This means that the body then has to keep these inflammatory processes regulated on top of ROS and other stress responses to training [187,190]. Hard training blocks also take their toll on the body and make it more vulnerable to infections. Training load should always be individualized and only carefully increased, keeping a close eye on the training response of the athlete. An overload of training is also linked to increased gut permeability and altered immune function [169] that can also be connected to diet, stress or deficiencies.

A supplementation with probiotics is definitely worth considering, as research has proven multiple times that lactic-acid bacteria can help hinder the outbreak and reduce severity of URTI [136]. And as the gut biome adds to the reduction of ROS, research has investigated bacterial strains including *Lactobacillus fermentum*, *Lactococcus lactis*, *Lactiplantibacillus plantarum*, *Lactobacillus gasseri*, and *Streptococcus thermophilus* that increase superoxide dismutase (SOD) activity [191]. Vitamin D has been identified as a modulating factor in maintaining immune homeostasis in healthy individuals via adjustments to the composition of the microbial community in the gut [188]. As hypothesized, supplementation with vitamin D stimulates the growth of good bacteria to preserve microbial immune homeostasis [82]. Supplementation with vitamin D should therefore be considered expressly in illness prone athletes. Zinc is also known for its modulatory functions of the immune system [192] and can be beneficial for athletes in intensive training blocks, especially when prone to URTI [126].

### 3.5. Targeting Gut Barrier Function

Gut permeability is a pivotal topic for elite athletes that have strenuous training routines. It is linked to many other issues that come with increased gut permeability. Immune function is one problem, GI problems and systemic inflammation another. Diet is essential in maintaining gut barrier function and should therefore be looked at first, together with the training routine. 

Further advantages can be gained by supplementation with probiotics, either used on a regular basis or as needed. As one study showed, taking a *Lactobacillus* supplement prior to an endurance event could speed up the recovery of the microbiota [117]. Speeding up the recovery of the microbiota could also mean faster recovery for the athletes, as the microbiota has the ability to help recovery in many ways, such as the modulating the anti-inflammatory response. The *Actinobacteria* genus, like *Bifidobacterium* or *Collinsella*, can support intestinal epithelial function via an immunomodulatory and anti-inflammatory function [193]. The effectiveness of probiotic supplementation is most likely based on enhanced SCFA production, increased mucus production, a lowered intestinal pH, an increased barrier function, and higher immune cell activity [2,194].

### 3.6. Targeting Cognition

Being in the right head space in a training session is vital when operating at an elite level. Many sport modalities demand a high level of coordination, quick thinking, and decision making, resulting in mental fatigue [195]. All previous targets need to be aligned to meet that standard. 

Coping with stress is a task of its own. Being elite in the sporting world, stress can often exceed the level that can be handled [196]. Stress response is linked to the gut microbiota, which can be explained by the gut-brain-axis [114]. Keeping stress at a balanced level benefits the gut biome, as it reacts to stress with a shift in the microbial community. Taking an approach that focuses on psychology can be suggested at this point. Limiting stress through a structured routine and scheduled time off, plus practicing stress release methods, such as breathing exercises and mediation, could be advantageous. 

### 3.7. Targeting Specific Female Athlete Requirements

Including a focus on female athletes is something that should be part of an individualized training approach. Female athletes differ from male athletes, therefore, training needs to be adapted to fit female physiology. The bi-directional connection between the gut microbiota and fluctuating female sex hormones [154] could impact performance as women are exposed to differing gastrointestinal symptoms. Nutrition and the fueling strategy should be adjusted to the individual state of the menstrual cycle and the impact of certain foods needs to be reconsidered. For the early detection of REDs, regularly dietary check-ups could be beneficial. Taking a gut-centric approach to support the prevention/treatment of REDs and the symptoms that come with them, the diet should be focused on an increased intake of complex carbohydrates. This not only ensures energy supply but might also counteract the reduction of carbohydrate fermenting microbes. A rebound in these bacteria could improve gastrointestinal symptoms through the production of SCFAs and by supporting gut homeostasis [163]. Additionally, intestinal microbes are involved in bone metabolism [164,165] and therefore need to be protected, to not worsen bone metabolism interference.

## 4. Practical Recommendations

**Figure 2:**
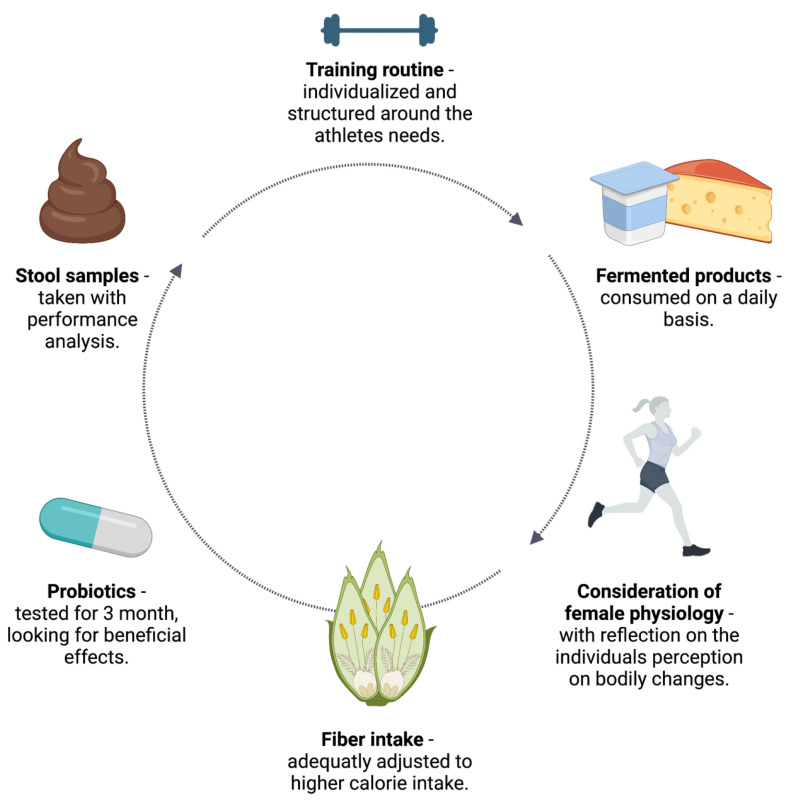
Practical recommendations aimed at ensuring athletes’ health and supporting performance with a focus on the gut microbiota. These practical applications can be adjusted immediately by the athlete and/or the coach. The emphasis lies in the practicability of the adjustments, which need to be tested firsthand. Created with BioRender.com.

The findings provide practical recommendations from which, when taken into practice, athletes could benefit. Figure 2 displays the practical recommendations.

### 4.1. Training Routine

Next to the positive effects and modulations that exercise brings to the gut microbiota, rising evidence shows the negative effects that a demanding training routine has on an athletes gut [8,165,169,192,193]. These unfavorable effects are likely to be traced back to altered gut transit and motility caused by physical and psychological stressors. Additional factors, such as intestinal injury and oxidative stress caused by repetitive ischemia reperfusion due to exercise, lead to gut dysbiosis. Furthermore, succinate, a metabolite that potentially causes gut inflammation, could be discovered in the feces of endurance runners [165].

To avoid negative side effects on the gut, the training routine should align with the physical and psychological capacity of the athlete. Training intensity and volume should be carefully increased, evaluating the training response, mood, sleep, diet, and stool. Questionnaires to monitor the subjective effect that training has on an athlete could be a first step into obtaining a better understanding of the athlete’s body and avoiding overtraining and negative consequences.

### 4.2. Stool Sample Analysis

Stool samples could help identify how a training block or a rest period affects the status of the intestinal microbial community. Identifying changes in the gut microbiota can be difficult since it is a relatively stable system. Therefore, we recommend including a stool analysis after a change in training focus or when comparing the on- and off-season. The analysis could be included in performance diagnostics, where examinations such as spiroergometric examinations are executed [197]. An additional blood analysis can help draw connections between the different variables and obtain a coherent image of the athlete. These blocks can vary in different sports due to diverse competition schedules and must be arranged around these. 

### 4.3. Probiotics

Human gut microbes are often compared to fingerprints, expressing their uniqueness in each one of us. Concluding that probiotics are not beneficial to everyone is easy to imagine. However, a proposal for the intake of probiotics has been made because there is evidence guiding us in that direction [117,125,127,137,194]. This is why supplementation that is accompanied by a physician and/or dietician could be worth considering [194]. The food industry suggests a minimum of one million colony-forming units (CFU) at the time of ingestion [198]. Studies investigated timeframes of four to sixteen weeks for supplementation [132,137,139,193]. We recommend a short time trial in the off-season, to see if complications arise and to get athletes into the routine of taking these supplements. Supplementation should carry on for up to three months—if positive effects fail to materialize, supplementation can be broken off. Specific foods also include probiotics as an alternative to supplements.

### 4.4. Fiber-Rich Foods

Fiber can help balance a diet rich in monosaccharides and high in protein [170,181], both of which are common in performance sports. Through a fiber-rich diet, the production of SCFAs can be increased, which has been demonstrated to be supportive for gut and overall health and may further support athletes’ performance. Therefore, athletes should have special interest in aiming for a diet that includes a variety of fiber-rich foods on a daily basis. Table 1 serves as an overview of foods and their fiber content [38,199]. 

### 4.5. Fermented Foods

Fermented foods are known for their ani-inflammatory properties. Athletes significantly challenge their immune system and could benefit from the additional consumption of fermented foods that support the inflammatory response [175,196]. Consuming them daily and adding them to a balanced diet could have positive effects on athletes’ gut microbiota. Examples of fermented foods are listed in Table 2 [200]. Lactic acid bacteria (L.A.B.) are the main microorganisms involved in fermentation and are critical for the benefit of fermented products. Different bacteria are needed for the fermentation of each food/food group, for example *Lactobacillus bulgaricus* in dairy products, *L. pantheris* in cereals, or *Leuconostoc mesenteroides* for vegetable products [9]. 

## 5. Future Directions

Future studies should focus on randomized and controlled trials using combined modern analytical methods to gain further insights into the importance of specific constituents, bacteria as well as metabolites. This should include basic scientific studies as well as studies in humans, optimally also involving athletes. There should also be special focus on the possibilities of influencing the microbiota so that interventional options can also be explored. 

## 6. Conclusions

The human gut microbiota is a complex physiological system and is linked to individual athlete performance through various different levels. In addition to its importance for general health maintenance, it shows bidirectional interactions with energy metabolism, muscular performance, immune function, intestinal epithelial integrity, and cognitive processes in sport. Looking at microcycles, it seems to be a rather stable system which can, however, be adapted over time by specific measures. These include primarily nutritional measures as well as training modalities and cognitive strategies. Accordingly, the inclusion of microbiota analysis offers opportunities to monitor and, if necessary, influence long-term health maintenance and performance development. 

The fact that there is insufficient data on elite sport and therefore much has to be deduced is certainly a limiting factor. Furthermore, cross-sectional studies do not always allow causalities to be established between the structure of the microbiome and the potential influencing factors.

## Figures and Tables

**Table 1:** Fiber rich foods.

Food (Serving Size)	Dietary Fiber (g/Serving)
Peas, cooked (½ cup)	8.1
Lentils, cooked (½ cup)	7.8
Figs, dried (2)	4.6
Wheat bran flakes (¾ cup)	4.6
Kidney beans, canned (½ cup)	4.5
Pear (1)	4.0
Apple, with skin (1)	3.7
Brown rice, cooked (1 cup)	3.5
Oatmeal, cooked (¾ cup)	3.0
Mixed nuts, roasted (1 handful)	2.6
Whole wheat bread (1)	1.9

**Table 2:** Examples of fermented foods.

Food Group	Examples of Foods
Dairy	Cheese, yoghurt, kefir, lassi, yoghurt
Beverages	Beer, kombucha, wine, cider
Soy	Soy sauce, soy yoghurt, soy milk, miso
Fruits & vegetables	Pickled fruits and vegetables, olives, kimchi, sauerkraut
Cereals	Bread and bakery products

## Data Availability

Not applicable.
